# A Systematic Review of Flywheel Training Effectiveness and Application on Sport Specific Performances

**DOI:** 10.3390/sports11040076

**Published:** 2023-03-30

**Authors:** Andrea Buonsenso, Marco Centorbi, Enzo Iuliano, Giulia Di Martino, Carlo Della Valle, Giovanni Fiorilli, Giuseppe Calcagno, Alessandra di Cagno

**Affiliations:** 1 Department of Medicine and Health Sciences, University of Molise, 86100 Campobasso, Italy; 2 Faculty of Psychology, eCampus University, 22060 Novedrate, Italy; 3 Department of Movement, Human and Health Sciences, University of Rome “Foro Italico”, 00135 Rome, Italy

**Keywords:** eccentric overload training, flywheel, iso-inertial, sports

## Abstract

The objective of this systematic review was to examine the effectiveness of flywheel training, which allows for the replication of specific sports movements, overloading both the concentric and eccentric phases. Inclusion criteria were injury prevention outcomes; ability in terms of strength, power, sprinting, jumping and change of direction; competitive athletes; and RCTs. Exclusion criteria were a lack of a control group and lack of baseline and/or follow-up data. The databases used were Web of Science, Scopus, PubMed, Cochrane Library, and Sage. The revised Cochrane risk-of-bias tool was used to assess the quality of the selected RCTs. The Oxford Centre for Evidence-Based Medicine 2011 Levels of Evidence was used. A PICOS (participants, intervention, comparators, study outcomes, and study design) approach was used to evaluate eligibility. A total of 21 RCTs with 8 to 54 participants in each study analyzed flywheel technology and its application in nine sports. The results showed that flywheel training is a good strategy to improve sports performance, providing variation in training methodologies and athletes’ adherence. Further studies are needed to define guidelines on training modality, weekly frequency, volume, and inertia load. Only a few studies have applied the flywheel device directly to overload specific multidirectional movements at different joint angles. This method is not exempt from criticalities, such as the cost and the ability to carry out only individual training.

## 1. Introduction

Technology has radically changed sports in all aspects. As demands for both amateur and professional sports have increased, science and technology have provided a significant advantage in monitoring and improving athletic performance [1]. The flywheel device could be an innovative training method to improve sport-specific performance by directly overloading both the concentric and eccentric phases of specific sport movements [2], ensuring a direct transfer to athletic performance.

**Figure 1:**
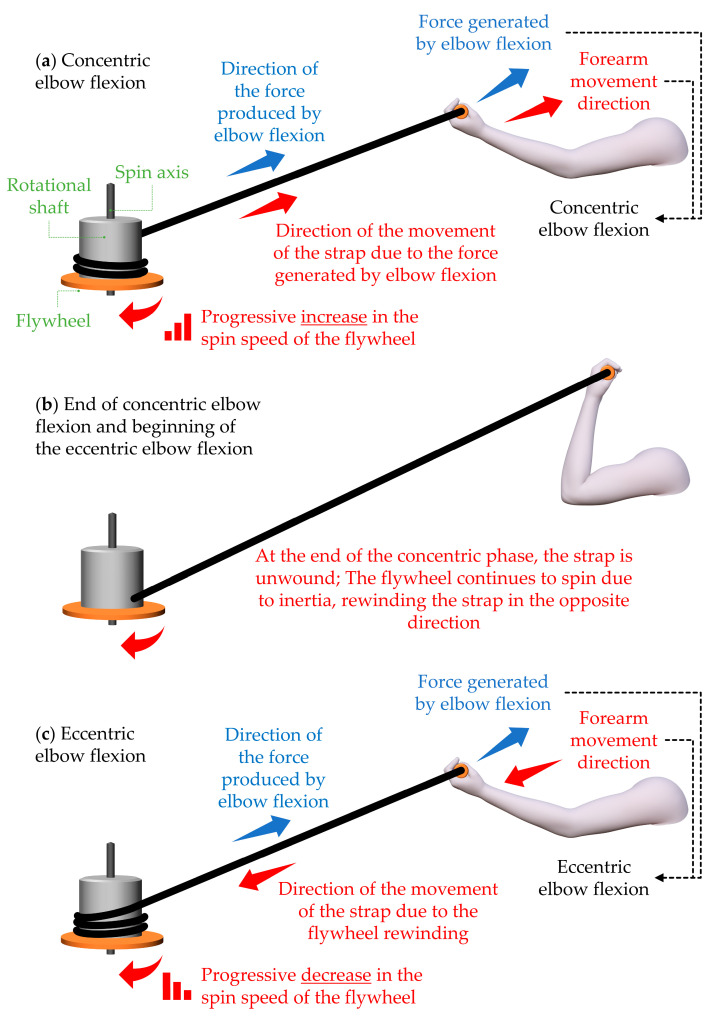
This figure shows a graphical illustration of a functioning flywheel device. An elbow flexion exercise was considered in the example: (**a**) concentric elbow flexion; (**b**) end of concentric elbow flexion and beginning of eccentric elbow flexion; (**c**) eccentric elbow flexion.

Berg and Tesch [3] developed the flywheel device to preserve astronauts’ muscle mass during space travel, providing resistance independently of gravity and weight. The device operates as follows: during the concentric phase of motion (acceleration), a strap attached to the flywheel completely unwinds, storing kinetic energy in the system. In the eccentric phase (deceleration), the system generates resistance as the strap is pulled back onto the shaft in response to the applied force until the flywheel comes to a stop [4]. A graphical illustration of a flywheel device is shown in Figure 1.

To use the device optimally, the athlete must apply maximum force during the acceleration phase to generate a resistance force proportional to their concentric effort in the eccentric phase. The resistance is, in fact, proportional to the acceleration produced during the concentric phase of the exercise. Since there is no resistance at a constant speed, flywheel exercises require a succession of accelerations and decelerations [4]. Flywheel technology allows for unlimited linear resistance loads during the concentric and eccentric muscular actions, with the ability to regulating the resistance loads during each repetition, determining maximum effort from the first repetition and throughout the entire range of motion [5]. This condition allows the subject to perform individualized load repetitions: since the force decreases with fatigue, the speed of the flywheel slows down accordingly. Therefore, the concept of maximum repetition does not exist in the flywheel training program because the exercise could virtually continue indefinitely, even with reduced force [4].

Several studies have shown that the prolonged and high eccentric strain caused by eccentric overload might lead to a preferential recruitment of high-threshold motor units [6]. This condition allows for the generation of greater maximal force/tension with a lower metabolic cost for the same work produced, maximizing muscle strength gains and regional hypertrophy and thus remodeling the muscular architecture [7].

Eccentric training has gained popularity due to its benefits for sports performance [8,9], rehabilitation [10,11], and reducing the risk of injury [12]. With regard to the effects of isolated eccentric overload on performance improvement, Chazaud [13] highlighted that greater muscle damage and inflammatory responses stimulate muscle protein synthesis and satellite cell differentiation. Annibalini et al. [14] showed that muscle damage induces early molecular and systemic muscle adaptations. Norrbrand et al. [15] showed that eccentric overload training produces greater electromyographic activity than traditional resistance training, leading to a higher mechanical load that promotes greater protein synthesis and, therefore, increased muscle hypertrophy. Walker et al. [16] reported that eccentric overload training increases maximum force production, increasing work capacity and reducing fatigue during training in trained subjects. In addition, the authors showed that it takes at least 5 weeks before these changes occur.

The eccentric overload that can be achieved during a workout is given by the inertial load used [17], the technique [18], and the previously applied concentric movement [19], independently of the acceleration due to gravity.

Additionally, as a new proposal, flywheel training allows for greater variation in training methodologies, and, consequently, promotes athlete adherence [20].

Several studies have demonstrated the effectiveness of this equipment, compared with other traditional unspecific training methods, in improving maximal isometric torque and isokinetic eccentric torque, sprint performance, and muscle strength [16,21,22]. Flywheel training’s effects in increasing the muscle cross-sectional area are similar to the results following traditional resistance training [16] and higher than for unspecific plyometric training [21].

Despite Raya-González et al. [2] claiming that the effectiveness of this methodology has not yet been well-established, several studies have confirmed its efficacy, showing that the combination of concentric and eccentric contraction leads to better adaptations when compared to only concentric or eccentric training [9,11]. This is probably due to the higher force expressed during the eccentric action, which maximizes the stretch-shortening cycle, allowing for greater force production during the subsequent concentric phase [23].

Several reviews have stated that the flywheel training methodology, using different protocols, can improve sprinting, change of direction (CoD), jump performance [2,24], power, and strength [25], even if applied in an unspecific manner. In addition, it has positive effects on muscle mass gain [10], maximum voluntary contraction [26] and 1-repetition maximum [23], and also reduces the risk of injury [27]. This is a key factor, considering its direct impact on the continuity of training and, consequently, on the improvement of sport performance. It has been shown that injury prevention programs improve soccer performance [28].

Nevertheless, this method is not exempt from criticalities, such as cost and the ability to carry out only individual training. On the other hand, the application of this device allows for the overloading of multidirectional movements in different joint angles and specific sport conditions, stimulating both the concentric and eccentric phases of muscle contraction. For this reason, this method can effectively contribute to the improvement of specific sports performance. Considering that, to our knowledge, other devices do not provide the same benefits, the purpose of this systematic review was to analyze randomized controlled trials that have evaluated the effectiveness of applying this method to athletes from different sports contexts, using both specific and nonspecific exercises.

## 2. Materials and Methods

### 2.1. Selection Criteria

A PICOS (participants, intervention, comparators, study outcomes, and study design) approach was used to evaluate the eligibility of the studies [29]. The inclusion and exclusion criteria are reported in Table 1.

### 2.2. Information Sources and Search Strategies

This systematic review was conducted according to the Preferred Recording Items for Systematic Review and Meta-analysis (PRISMA) statements [30]. In this systematic review, all randomized controlled trials (RCTs) dealing with the effects of flywheel eccentric overload training on athletic performances published in a peer-reviewed journal were considered. A systematic search was performed across Web of Science, Scopus, PubMed, Cochrane Library, Sage, and other databases where flywheel technology was employed to select peer-reviewed articles of interest published between 1994 (start date) and 2023. The search engine Google Scholar was also used for the systematic search of peer-reviewed articles. For each database, the following combinations of terms were used: “flywheel training” OR “eccentric overload training” OR “isoinertial training” AND “sports performance”; “flywheel training” OR “eccentric overload training” OR “isoinertial training” AND “injury prevention”; “flywheel training” OR “eccentric overload training” OR “isoinertial training” AND “strength”; “flywheel training” OR “eccentric overload training” OR “isoinertial training” AND “power”; “flywheel training” OR “eccentric overload training” OR “isoinertial training” AND “jump performance”; “flywheel training” OR “eccentric overload training” OR “isoinertial training” AND “sprint”; “flywheel training” OR “eccentric overload training” OR “isoinertial training” AND “change of direction”.

### 2.3. Selection and Data Collection Process

The search results were screened according to titles, abstracts, and full text by three researchers who worked independently. Additionally, the reference lists of the selected articles were screened for further relevant studies. In cases where there was a lack of consensus among the researchers, a fourth researcher was consulted to help resolve the disagreement.

### 2.4. Data Items

The authors of the present systematic review sought to examine several aspects related to enhancing sports performance through the application of flywheel devices in addition to traditional training, including improvements in strength, power, sprinting, jumping, change of direction, and injury prevention. The authors also selected studies that applied the device directly to sport-specific movements to enhance sports performance, overloading both the concentric and eccentric phases to ensure a direct transfer of strength improvements to athletic performance across various joint angles and specific multidirectional movements.

### 2.5. Study Risk of Bias Assessment

**Figure 2:**
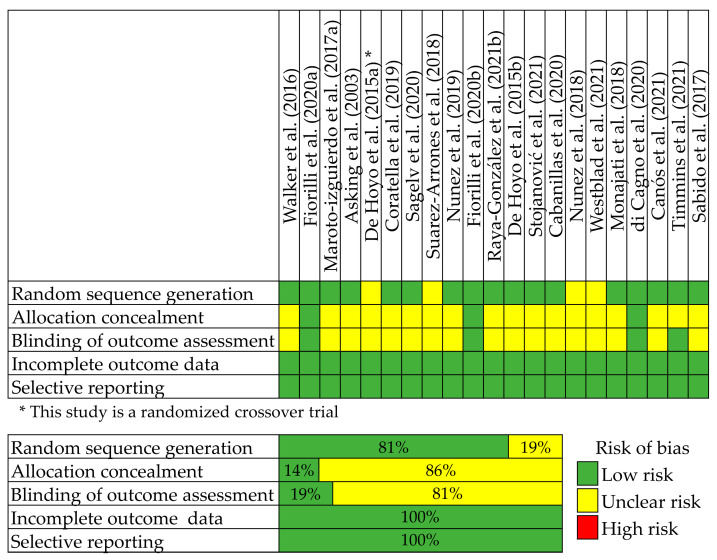
Risk of bias assessment. The blinding of participants and personnel was not assessed because the nature of the interventions did not allow for blinding of the participants and personnel [16,21,23,27,31,32,33,34,35,36,37,38,40,41,42,43,44,45,46,47,48].

The revised Cochrane risk-of-bias tool (RoB 2 tool) for randomized trials was used to assess the quality of the RCTs included in the study (Figure 2). The Oxford Centre for Evidence-Based Medicine 2011 Levels of Evidence (OCEBM) for the present paper described is 1a.

## 3. Results

**Figure 3:**
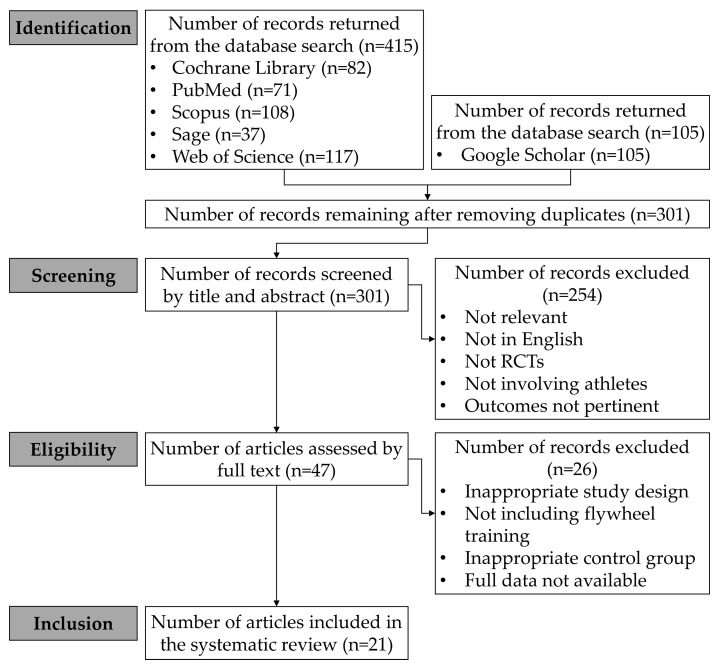
Flowchart of the study.

A total of 520 articles were initially selected. Of these, 219 were excluded as duplicates (selected in multiple databases). After the screening of the titles and abstracts, 254 articles were excluded from the analysis as they were not relevant to the aim of this review, were not in English, were not randomized controlled trials (RCTs), did not involve athletes, or measured outcomes that were not pertinent to the aim of this review. Subsequently, 26 studies were excluded after full-text analysis due to inappropriate study design/intervention, absence of flywheel training (e.g., eccentric training was performed using other types of devices/strategies), inappropriate/no control group, or lack of availability of baseline and/or follow-up assessments. A total of 21 RCTs were included in the systematic review. The study flowchart is shown in Figure 3.

### 3.1. Study Characteristics

**Figure 4:**
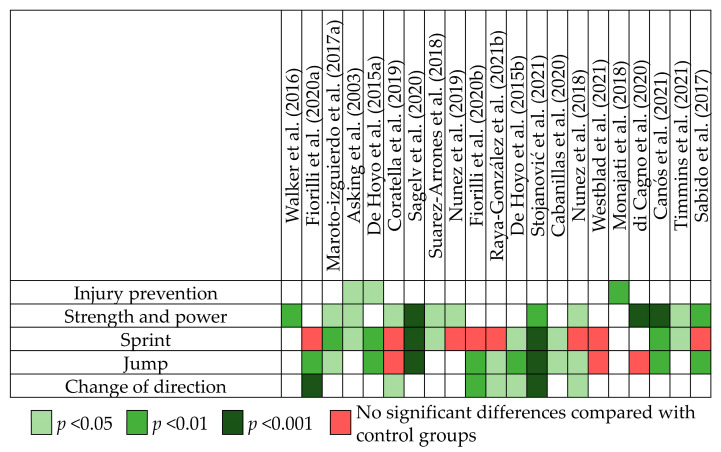
Main results of RCTs. The green cells indicate that the authors of the studies reported significant better results for flywheel training groups compared with the other groups. The red cells indicate instead an insignificant difference [16,21,23,27,31,32,33,34,35,36,37,38,40,41,42,43,44,45,46,47,48].

Twenty-one RCTs were analyzed in the systematic review. These studies applied flywheel technology to the training of nine sports: 10 studied soccer [21,27,31,32,33,34,35,36,37,38]; 2 studied handball [23,39], basketball [40,41] and team sports in general [42,43]; and 1 studied volleyball [44], fencing [45], tennis [46], football [47], and fitness [16]. The number of participants across the included studies ranged from 8 to 54, with all studies enrolling male participants except for two that involved both male and female participants [43,44]. The mean age of the participants was 19.25 ± 2.05 years, except for one study which did not report the mean age of participants who were under 16 [37]. The flywheel intervention lasted between 6 and 35 weeks, and the training frequency ranged between 1–3 sessions per week. Two studies evaluated the acute effects of a single session of flywheel training [36,38]. Fifteen studies applied the flywheel device in an unspecific manner [16,23,27,31,32,33,34,35,37,38,42,43,44,47,48], while only six applied it to specific sport movements [21,36,40,41,45,46]. For all RCTs included in this review, the performances were analyzed, and the main results are presented in Figure 4.

### 3.2. Flywheel Training and Injury Prevention

Only a few RCTs have evaluated the effects of flywheel training on injury prevention: two on soccer [27,31] and one on volleyball [44]. Several studies were excluded in the review [2,49,50] because they did not directly assess injury risk.

It is well established that strength training plays a fundamental role in preventing sport injuries and, in this regard, Askling et al. [27] suggested that strength training should include eccentric contractions to ensure adequate muscle responses. Therefore, flywheel training could be an appropriate choice. Several studies verified the efficacy of flywheel training in preventing injuries in different team sports. Regarding soccer, advantages following the combination of flywheel and traditional training were found in both amateur and elite players. Askling et al. [27] found a lower injury rate in elite male soccer players after 10 weeks of hamstring flywheel training compared to a traditional soccer training. De Hoyo et al. [31] also observed a lower injury frequency and severity in young elite soccer players after 10 weeks of eccentric flywheel overload training compared to the usual technical/tactical training, showing that the eccentric component of strength was improved [51].

Monajati et al. [44] evaluated the effects of 6 weeks of flywheel training on the performance of recreational volleyball players. The authors detected an enhancement in the tuck jump and a particular improvement in landing technique due to the enhancement of the neuromuscular control, which led to reduced anterior cruciate ligament injuries and lessened risk of injuries in general [52].

The applications of flywheel training for injury prevention are not currently well-explored in the female population [53].

Although few studies have investigated the effects of flywheel training on injury prevention in sports, some practical applications should be taken in consideration: training frequency at least two times per week for at least of 10 weeks, with emphasis on involvement of the squat and hamstring muscles in order to reduce lower limb injuries. The results of flywheel training on injury prevention are shown on Table 2.

### 3.3. Flywheel Training and Strength and Power

The benefits of flywheel training on physical capacities such as strength and power, in both individual and team sports, have recently been assessed in several reviews and RCTs. Two recent reviews [54,55] and several RCTs were considered: four on soccer [27,32,33,34] and one on football [47], basketball [40], handball [48], tennis [46], fencing [45], fitness [16] and team sport in general [42].

Flywheel training has generally been applied on the lower limbs, overloading squats and half squats to improve the concentric and eccentric strength of knee flexors, or isometric strength of the lower limbs in both professional or junior athletes. Only one RCT [46] applied flywheel training on the upper limbs of athletes practicing tennis in an unspecific manner, highlighting good results in terms of power, but no significant improvements in the specific sporting gesture (service).

Regarding strength, a 10-week flywheel training program was applied to semi-professional and professional soccer players, aiming to improve both the concentric and eccentric isokinetic strength of knee flexors [27,32]. Timmins et al. [47] reported a significant improvement in the eccentric strength of the knee flexors in semi-professional football players after 35 weeks of deadlift flywheel training.

Regarding improvement in the lower limb isometric strength, Stojanović et al. [40] reported significant results in junior basketball players after 8 weeks of flywheel training, and Walker et al. [16] showed improvements in both maximal isometric torque and isokinetic eccentric torque in strength-trained men after 10 weeks. In contrast, Sagelv et al. [33] reported improvement in maximal squat strength in amateur soccer players, with more significant results after a traditional squat protocol than a flywheel one.

Differences in maximum neural activation and recovery ability between training sessions of athletes of different technical levels may explain the differences in strength outcomes [54]; in addition, it is possible to conclude that well-trained athletes may be able to reap greater benefits from this training than low-trained athletes.

Regarding power development, in team sport athletes, Nunez et al. [42] reported significant improvement in half-squat and lateral lunge power after 6 weeks, and Sabido et al. [48] reported a higher power output in both the eccentric and concentric phases of the half squat in handball players. In soccer, Suarez-Arrones et al. [34] applied flywheel training on elite players during the entire competitive season (27 weeks), with significant improvement in half squat power output. Maroto-Izguierdo et al. [23] showed enhancement in muscle power output at different submaximal loads after 6 weeks of flywheel training in handball players. Nunez et al. [35] reported an improvement in eccentric mean power and in the eccentric/concentric ratio after 9 weeks of flywheel training in elite soccer players.

Referring to individual sports, Canós et al. [46] evaluated the effect of 8 weeks of flywheel training in young tennis players and reported significant improvements in forehand medicine ball throws, but not in serve velocity. The authors hypothesized that this study period was insufficient to reflect improvements in service velocity. Six weeks of flywheel training applied to the specific sport movements (lunge and fencing assault) of young elite fencers led to a significant improvement in the movement speed [45].

In conclusion, 6–10 weeks of flywheel training two times per week, seems to be a good strategy to improve strength and power more significantly than traditional methods of strength conditioning. However, these benefits seem to differ depending on the type of exercise [24] and the athlete’s training experience [56]. The results of flywheel training on strength and power performance are shown in Table 3.

### 3.4. Flywheel Training and Sprint

Conflicting results on the effects of flywheel training on sprint performance are reported in the literature [24,37]. The results are influenced by the different applications of flywheel training, training frequency, and years of experience of the athletes [37], as well as tests performed with different typologies (standing vs. flying) [54]. Most of the studies were carried out on athletes involved in team sports and particularly focused on changes of direction: ten RCTs focused on soccer [21,27,31,33,38]; two on handball [23,57], basketball [40,41], and team sport in general [42,43]; and one on football [47] and tennis [46].

Flywheel training resulted to be more efficient than traditional strength training (weight training) in improving 20 m sprint time in professional handball players, even after 15 flywheel training sessions [23]. Conflicting results were found regarding the efficacy of this training modality on sprinting performance: several studies reported no improvement of sprint performance in semi-professional and young soccer players [42,57] and team sport players [42,57]. Coratella et al. [32] showed no significant effect on 10 m and 30 m sprint performance after 8 weeks of squat flywheel training. Fiorilli et al. [21] showed significant improvement in the athletes’ 60 m linear sprint performance after 6 weeks of flywheel training in soccer players, but there was no difference compared to traditional soccer training. Canós et al. [46] reported significant enhancement in 10 m sprint time after 4 weeks of eccentric overload training in young tennis players; while this performance decrease from week 4 to week 8 measurements. This could be due to fatigue induced by an inadequate management of training loads and recovery time, which affect the production of strength and neural control [57].

Some authors stated that a long period of application is required to achieve significant improvements: Suarez-Arrones et al. [34] reported significant improvement in linear sprint performance after 27 weeks of flywheel training, while Timmins et al. [47] showed an improvement of maximal sprint performance in semi-professional football players after a long period (35 weeks) of twice weekly flywheel training. Conversely, De Hoyo et al. [38] reported significant improvement in sprint performance after a single bout of flywheel training, and Fiorilli et al. [36] showed improvement in the 40 m sprint test after a single bout of flywheel warm-up in young soccer players, with similar results compared to a traditional warm-up.

Concerning improvements related to short-, middle-, and long-distance sprinting, Sagelv et al. [33] showed significant improvements in 10 m sprint time after 6 weeks of flywheel squat training. De Hoyo et al. [31], after 10 weeks of quadricep and hamstring flywheel training in elite soccer players, reported an improvement in the 10 to 20 m sprint test, but a lack of improvement in the 0 to 10 m sprint, likely due to the fact that the hamstring muscles are more involved in long-distance sprints than in shorter ones. In support of these results, Askling et al. [27] reported a significant improvement in 30 m sprint performance after eccentric overload training of the hamstrings; however, this improvement was lower in the first 20 m than that found in the 30 m. Cabanillas et al. [41] showed significant improvements in the 30 m sprint performance after 6 weeks of half squat flywheel training in basketball players. In contrast, Stojanović et al. [40] reported significant improvement in the 5 m sprint test, but not in the 20 m test, in junior basketball players after a total of 12 eccentric overload training sessions. The authors justified this result by highlighting that 20 m sprints are rarely observed during basketball games, while sprints of 5/10 m are more specific to basketball performance [58].

Since the literature has shown conflicting results in terms of performance improvement using this method, further studies are needed to define the effectiveness of flywheel training in improving sport performance. The results of flywheel training on sprint performance are shown on Table 4.

### 3.5. Flywheel Training and Jump Performance

Several reviews [54,55] and RCTs have highlighted the efficacy of flywheel training in improving jumping performance. Elastic potential energy, which is developed during the stretch-shortening cycle, may be a key factor in improving this performance [59].

Seven RCTs focused on soccer [21,31,32,33,36,37,38,60]; two on handball [23,48], basketball [40,41] and team sports in general [42]; and one on football [47]. Only one study focused on an individual sport (tennis) [46].

Almost all the studies confirmed positive results for the counter movement jump (CMJ), drop jump (DJ), and hopping tests in different samples of athletes who followed different training protocols.

Coratella et al. [32] demonstrated significant improvements in squat jump (SJ) and CMJ performance, but with no significant differences with the control group, in semi-professional soccer players after 8 weeks of flywheel squat training. De Hoyo et al. [31] reported an improvement in CMJ performance after 10 weeks of quadricep and hamstring flywheel training in elite soccer players. Significant improvements were found after 6 weeks of training, twice a week, in CMJ performance in 38 amateur soccer players [33] and in DJ and repeat hop performance in youth soccer players [21]. Stojanović et al. [40] found a significant improvement in CMJ in junior basketball players after 8 weeks of training, highlighting better improvements than the control group. The same result was found by Cabanillas et al. [41]. Nunez et al. [42] reported significant improvements in CMJ after 6 weeks of unilateral and bilateral flywheel training. Westblad et al. [43] showed improvements in squat jump performance after 6 weeks of flywheel squat training, but with no difference compared to traditional strength training. Maroto-izguierdo et al. [23] showed significant improvement in CMJ and SJ in professional handball players after 15 flywheel training sessions. Canós et al. [46] found significant improvements in CMJ performance after 8 weeks of flywheel training in tennis players; this improvement was greater in the first 4 weeks, and was maintained in the next 4 weeks. Sabido et al. [48] showed significant improvements in specific triple hop distance performance in handball players after adding a single flywheel training session per week to their traditional resistance training. In addition, Fiorilli et al. [36], showed that flywheel warm-ups enhanced explosive and reactive strength, as assessed by SJ, CMJ, and DJ, more that a traditional warm-up in young soccer players. The same result was found by De Hoyo et al. [38] in CMJ performance after a single bout of flywheel training.

Conversely, a few studies failed to show significant results after flywheel training in soccer [25,54] and fencing [45], probably due to the lack of specificity between the test and the training protocol [2,61].

In conclusion, the literature recommends a training frequency of 2/3 times per week to improve jump performance [23,56]; however, most of the RCTs analyzed herein achieved significant improvements in this performance even with one session per week. The results of flywheel training on jump performance are shown on Table 5.

### 3.6. Flywheel Training and CoD

Several reviews have highlighted the effectiveness of flywheel training in enhancing CoD performance [2,62,63]. Five RCTs focused on soccer [21,32,36,37,38,60], and one on basketball [40] and team sports in general [42]. The similitude between CoD tasks and flywheel movement could justify this enhancement [60]. Several factors influence CoD performance, the most important being the eccentric strength of the thigh muscles [64]. The eccentric strength influences the deceleration phase and can facilitate re-acceleration in a different direction during CoD performance [65]. The brake impulse generated by the flywheel allows for more elastic energy storage, which contributes to greater force output during CoD performance [66].

Nunez et al. [42] reported greater improvements in CoD turns of 90° after 6 weeks in the experimental group, which performed unilateral, compared to bilateral, flywheel training. 

Regarding young soccer players, several authors [21,32,37,38] found significant improvements in both contact time and squats during CoD performance after 10–8 and 6 weeks of flywheel training, respectively. In addition, Fiorilli et al. [36], directly overloading the CoD task, showed significant improvements in CoD performance. This was evaluated with the Illinois agility test after a warm-up performed on a flywheel device, highlighting that the enhanced effects of this type of training on CoD performance persisted up to 10 min after the administration of the warm-up.

Stojanović et al. [40] showed significant improvements in CoD tasks after 8 weeks of flywheel training in male basketball players compared with traditional basketball training.

In conclusion, all of the analyzed RCTs showed improvements in athletes’ performance following flywheel training. Training once or twice per week were shown be effective in improving CoD performance through the application of a flywheel device. These results are shown in Table 6.

### 3.7. Unknown Overload of Flywheel Training and Performance

Few studies have examined the effects of training with unknown or unpredictable loads, such as flywheel training [67]. Training with an unknown load seems to significantly improve multi-joint stretch–shortening cycle movement, and may elicit central neural adaptations which increase muscle activity and speed of contraction. Moreover, this type of training may improve performance in multi-joint movements that require a stretch–shortening cycle [67]. The absence of knowledge regarding the eccentric overload applied by the device stimulates adaptations of the neuromuscular system, as well as intra- and intermuscular coordination [39]; Sabido et al. [39] showed greater gains in power output and throwing velocity after training with unknown loads in handball players. As team and individual sports are characterized by uncertain loads, athletes can benefit from flywheel training, as it allows them to apply similar movements and loads as those they would apply in competition [45]. It has been shown that both agonist [68] and antagonist muscles increase their activity in response to uncertain stimuli, which may be due to a neural adaptation that stiffens joints by increasing muscle co-contraction [69,70]. Hernández-Davó et al. [39] showed increased power output and muscle activation during maximal concentric contractions after training with uncertain loads.

## 4. Discussion

This review provides information on the use of flywheel training to improve specific sports performance. We also investigated several aspects of sport performance improvements, such as injury prevention, strength and power, sprinting, jumping, and CoD. We studied the application of the exercise in terms of on overloading both the concentric and eccentric phases of sport movements, with unknown overloads for each repetition.

This training method is widely used in sports. The application of flywheel devices in soccer performance training is currently the most extensively studied. Considering the high risk of injuries during CoD, kicks, and stops [71], most of them located in the lower limbs (quadriceps, hamstrings, calves, and adductors), the application of flywheel training may represent a good prevention tool. Additionally, in other sports, such as tennis, basketball, volleyball, and handball, there are an increasing number of studies emerging on the application of this method, intending not only to improve athletic performance, but also to reduce the risk of injuries.

Coordinative performance improves with age, and is positively influenced by practicing sports activities [72]. The unknown load of flywheel training stimulates inter- and intramuscular coordination, thus improving performance, especially in young athletes, who are of a better age to develop coordination. Moreover, flywheel protocols, requiring low training volume and frequency, are particularly recommended for athletes during in-season training to improve athletic performance [73].

Regarding the acute improvements obtained during the flywheel warm-up, it should be hypothesized that this effect could be due to a sort of post-activation potentiation (PAP) [74] that enhances neuro-motor performance [75]. Flywheel exercises should be a valid training strategy to obtain both acute performance enhancement and chronic adaptations [24,25]; nevertheless, it could still be advisable to combine flywheel and traditional resistance training to optimize athletic performance [24,76].

Flywheel training causes maximum muscle activation in the concentric phase and in part of the eccentric phase for each repetition, unlike traditional weight training, which requires maximum activation only during the “sticking point” [15]. Wonders [77] showed that flywheel training allows for optimal muscle strength production throughout the range of motion.

Although the effects of this training methodology are comparable to those of traditional ones, we suggest that flywheel training be included in sport periodization to diversify the training stimuli for athletes. Moreover, further studies are needed to better define guidelines on training modalities, such as weekly frequency, volume, and inertia load.

Despite the main advantage of flywheel device being the possibility to directly overload specific sports movements, most of the analyzed studies applied the device to non-specific movements coded for other traditional methods. Only a few studies have exploited this potential by applying flywheel devices in fencing [45], soccer [21,36], tennis [46], and basketball performance [40,41]. Since the results of these studies are promising and highlight significant improvements in specific sport performance, further studies on the application of flywheel training in other sports should be encouraged. We expect that future studies will evaluate its usefulness in specific movements of other sports, such as combat sports, overloading roundhouse kick in taekwondo, or boxing shots (such as jab, hook, and uppercut).

A potential limitation of this systematic review is that it only included studies written in English. We are aware that this is not in line with international recommendations [78]. Moreover, concerns could be raised regarding the inclusion criteria related to the RCTs, which could limit our knowledge of flywheel application.

## 5. Conclusions and Practical Applications

Flywheel training is a safe and time-effective strategy to enhance physical outcomes, allowing for multidirectional replication and overloading of specific sports movements with a lower metabolic cost. In addition, it allows for optimal muscle strength production throughout the range of motion. Flywheel training can improve athletic performance in terms of strength, power, sprinting, jumping, and CoD. The literature suggests that at least two training sessions per week are needed to improve sport performance. However, information on quantifying volume, intensity, and load may be diversified to achieve training goals in different sports and at different technical levels in the future. It is advisable to include flywheel training in sport periodization in order to diversify the training stimuli for the athlete.

## Figures and Tables

**Table 1:** Selection criteria.

Category	Inclusion Criteria	Exclusion Criteria
Population	Athletes	Non-athletes
Intervention	Flywheel training	Other training methodologies
Comparator	Active control group	Absence of active control group
Outcome	Measure of sport performance (injury prevention, strength, power, sprint, jump, change of direction)	Lack of baseline and/or follow-up data
Study design	Randomized controlled trials	All other study designs

**Table 2:** Effects of flywheel training on injury prevention.

Authors (Reference No.)	Sport and Sample	Training Performed	Duration and Frequency of Training Sessions	Specific or Not Specific Sport Movement	Main Results Obtained
Asking et al. (2003) [27]	30 male soccer players aged 25 ± 3.1	Leg curl in prone position (4 sets with 8 repetitions)	10 weeks during preseason (1–2 session per week, for a total of 16 training)	Non-specific sport movement	Lower hamstring strain injuries compared with control group during the entire season
De Hoyo et al. (2015a) [31]	36 male soccer players aged 17 ± 1	Half squat and leg curl in prone position (3–6 sets with 6 repetitions)	10 weeks (1–2 per week)	Applied to non-specific sport movement	Lower injury frequency and severity compared to control group
Monajati et al. (2018) [44]	10 male and female volleyball players aged 21.8 ± 3.74	Squat; single leg squat; straight leg deadlift; leg curl; lunges; hip extension (2 sets with 8 repetitions)	6 weeks (2 per week)	Applied to non-specific sport movement	Enhancement in the tuck jump, leading to an improvement in landing technique by reducing the knee valgus.

**Table 3:** Effect of flywheel training on strength and power performance.

Authors (Reference No.)	Sport and Sample	Training Performed	Duration and Frequency of Training Sessions	Specific or Not Specific Sport Movement	Main Results Obtained
Walker et al. (2016) [16]	33 male athletes practicing strength training fitness aged 22 ± 3	Leg press and unilateral knee extension (3 sets with 6–10 repetitions)	10 weeks (2 sessions per week)	Non-specific sport movement	Improvement in maximal isometric torque and isokinetic eccentric torque
Asking et al. (2003) [27]	30 male soccer players aged 25 ± 3.1	Leg curl in prone position (4 sets with 8 repetitions)	10 weeks during preseason (1–2 sessions per week, for a total of 16 training)	Non-specific sport movement	Improvement of concentric and eccentric isokinetic strength of knee flexors
Coratella et al. (2019) [32]	40 male soccer players aged 23 ± 4	Squat (4–6 sets with 8 repetitions)	8 weeks (1 session per week)	Non-specific sport movement	Improvement of concentric and eccentric isokinetic strength of knee flexors, but no difference with control group
Sagelv et al. (2020) [33]	38 male soccer players aged 23.87 ± 2.55	Squat (3–4 sets with 4–6 repetitions)	6 weeks (1 session per week)	Non-specific sport movement	Improvement in maximal squat strength, more significant than traditional squat protocol
Suarez-Arrones et al. (2018) [34]	14 male soccer players aged 17.5 ± 0.8	10 exercises for upper body and core, 10 exercises for lower body (1–2 sets with 10–12 repetitions)	27 weeks (entire competitive season, 2 sessions per week)	Non-specific sport movement	Improvement in half-squat power output
Stojanović et al. (2021) [40]	36 male basketball players aged 17.58 ± 0.5	Romanian deadlift and half squats (2–4 sets with 8 repetitions)	8 weeks (1–2 sessions per week, for a total of 12 training sessions)	Specific and non-specific sport movement	Improvement in lower limb isometric strength
Nunez et al. (2018) [42]	27 male athletes practicing team sports aged 22.7 ± 2.8	Unilateral lunge and bilateral squat (4 sets with 7 repetitions)	6 weeks (2 sessions per weeks)	Non-specific sport movement	Improvement in half-squat and lateral lunge power
di Cagno et al. (2020) [45]	54 male fencers aged 17.4 ± 2.3	Forward and backward lunge, two steps forward and backward, simulating the fencing assault (3–4 sets with 7–9 repetitions)	6 weeks (2 sessions per week)	Non-specific sport movement	Improvement in the lunge distance and fencing assault, maintaining the same execution time
Canós et al. (2021) [46]	24 male tennis players aged 15.7 ± 1.1	Low row 90°, forehand closed stance, backhand closed stance, one-handed chest crossover, one-handed low row, chest press, one-handed shoulder press (3 sets with 6–8 repetitions)	8 weeks (2 sessions per week)	Specific and non-specific sport movement	Improvement in forehand medicine ball throws, but not in serve velocity
Timmins et al. (2021) [47]	27 male football players aged 22 ± 3	Nordic hamstring and deadlift (5 sets with 3 repetitions)	35 weeks (2 sessions per week)	Non-specific sport movement	Improvement in eccentric strength of knee flexors
Sabido et al. (2017) [48]	18 male handball players aged 23.9 ± 3.8	Bilateral half squat (4 sets with 8 repetitions) and unilateral lunge (2 sets with 8 repetitions)	7 weeks (1 session per week)	Non-specific sport movement	Higher power output in eccentric and concentric phase of the half squat

**Table 4:** Effects of flywheel training on sprint performance.

Authors (Reference No.)	Sport and Sample	Training Performed	Duration and Frequency of Training Sessions	Specific or Not Specific Sport Movement	Main Results Obtained
Fiorilli et al. (2020a) [21]	34 male soccer players aged 13.28 ± 1.0	Multidirectional 4 m sprint, shot movement (4 sets with 7 repetitions)	6 weeks (2 sessions per week)	Specific sport movement	Improvement in sprint performance, but no difference compared to control group
Maroto-izguierdo et al. (2017a) [23]	29 male handball players aged 21.8 ± 1.3	Leg press (4 sets with 7 repetitions)	6 weeks (2–3 sessions per week, for a total of 15 training session)	Non-specific sport movement	Significant improvement in 20 m sprint time compared to traditional weight training
Asking et al. (2003) [27]	30 male soccer players aged 25 ± 3.1	Leg curl in prone position (4 sets with 8 repetitions)	10 weeks during preseason (1–2 sessions per week, for a total of 16 training)	Non-specific sport movement	Improvement in 30 m sprint performance
De Hoyo et al. (2015a) [31]	36 male soccer players aged 17 ± 1	Half squat and leg curl in prone position (3–6 sets with 6 repetitions)	10 weeks (1–2 sessions per week)	Applied to non-specific sport movement	Improvement in 0 to 20 m and 10 to 20 m in sprint test compared to control group
Coratella et al. (2019) [32]	40 male soccer players aged 23 ± 4	Squat (4–6 sets with 8 repetitions)	8 weeks (1 session per week)	Non-specific sport movement	No improvement in 10 m and 30 m sprint performance
Sagelv et al. (2020) [33]	38 male soccer players aged 23.87 ± 2.55	Squat (3–4 sets with 4–6 repetitions)	6 weeks (1 session per week)	Non-specific sport movement	Improvement in 10 m sprint time
Suarez-Arrones et al. (2018) [34]	14 male soccer players aged 17.5 ± 0.8	10 exercises for upper body and core, 10 exercises for lower body (1–2 sets with 10–12 repetitions)	27 weeks (entire competitive season, 2 sessions per week)	Non-specific sport movement	Improvement in linear sprint performance
Nunez et al. (2019) [35]	20 male soccer players aged 17 ± 1	Front step (2–3 sets with 6 repetitions)	9 weeks (1 session per week)	Non-specific sport movement	Improvement in 20 m sprint performance, but no difference compared to control group
Fiorilli et al. (2020b) [36]	12 male soccer players aged 13.3 ± 0.7	4 m multidirectional sprint (4 sets with 10 repetitions)	Single session (acute)	Specific sport movement	Improvement in 40 m sprint performance, but no difference compared to control group
Raya-González et al. (2021b) [37]	20 male soccer players with age <16	Lateral squat (2–4 sets with 8–10 repetitions)	10 weeks (1 session per week)	Non-specific sport movement	No significant variation in 10–20 m and 30 m linear sprint tests
De Hoyo et al. (2015b) [38]	20 male soccer players aged 17.0 ± 1.0	Half squat (4 sets with 6 repetitions)	Single session (acute)	Non-specific sport movement	Significant improvement in contact time and force during change of direction task.
Stojanović et al. (2021) [40]	36 male basketball players aged 17.58 ± 0.5	Romanian deadlift and half squat (2–4 sets with 8 repetitions)	8 weeks (1–2 sessions per week, for a total of 12 training session)	Specific and non-specific sport movement	Improvement in 5 m sprint test, but not in 20 m
Cabanillas et al. (2020) [41]	8 male basketball players aged 21.3 ± 3.45	Half squat (4–6 sets with 10 repetitions)	6 weeks (1 session per week)	Specific and non-specific sport movement	Improvement in 30 m sprint performance
Nunez et al. (2018) [42]	27 athletes practicing team sports aged 22.7 ± 2.8	Unilateral lunge and bilateral squat (4 sets with 7 repetitions)	6 weeks (2 sessions per weeks)	Non-specific sport movement	No improvement in 10 m sprint performance
Westblad et al. (2021) [43]	25 male and female athletes practicing team sports aged 11.8 ± 0.9	Squat (4 sets with 6 repetitions)	6 weeks (2 sessions per week)	Non-specific sport movement	No significant improvement in 10–20 m and 30 m sprint performance
Canós et al. (2021) [46]	24 male tennis players aged 15.7 ± 1.1	Low row 90°, forehand closed stance, backhand closed stance, one-handed chest crossover, one-handed low row, chest press, one-handed shoulder press (3 sets with 6–8 repetitions)	8 weeks (2 sessions per week)	Specific and non-specific sport movement	Enhancement in 10 m sprint time during the first 4 weeks; this performance decreased from week 4 to week 8
Timmins et al. (2021) [47]	27 male football players aged 22 ± 3	Nordic hamstring and deadlift (5 sets with 3 repetitions)	35 weeks (2 sessions per week)	Non-specific sport movement	Improvement of maximal sprint performance
Sabido et al. (2017) [48]	18 male handball players aged 23.9 ± 3.8	Bilateral half squat (4 sets with 8 repetitions) and unilateral lunge (2 sets with 8 repetitions)	7 weeks (1 session per week)	Non-specific sport movement	No improvement in sprint performance

**Table 5:** Effect of flywheel training on jump performance.

Authors (Reference No.)	Sport and Sample	Training Performed	Duration and Frequency of Training Sessions	Specific or Not Specific Sport Movement	Main Results Obtained
Fiorilli et al. (2020a) [21]	34 male soccer players aged 13.28 ± 1.0	Multidirectional 4 m sprint, shot movement (4 sets with 7 repetitions)	6 weeks (2 sessions per week)	Specific sport movement	Improvement in squat jump, drop jump, countermovement jump, and repeat hop performance
Maroto-izguierdo et al. (2017a) [23]	29 male handball players aged 21.8 ± 1.3	Leg press (4 sets with 7 repetitions)	6 weeks (2–3 sessions per week, for a total of 15 training session)	Non-specific sport movement	Improvement in squat jump and countermovement jump performance
De Hoyo et al. (2015a) [31]	36 male soccer players aged 17 ± 1	Half squat and leg curl in prone position (3–6 sets with 6 repetitions)	10 weeks (1–2 sessions per week)	Applied to non-specific sport movement	Improvement in countermovement jump performance compared to control group
Coratella et al. (2019) [32]	40 male soccer players aged 23 ± 4	Squat (4–6 sets with 8 repetitions)	8 weeks (1 session per week)	Non-specific sport movement	Improvements in squat jump and countermovement jump performance, but with no difference with control group
Sagelv et al. (2020) [33]	38 male soccer players aged 23.87 ± 2.55	Squat (3–4 sets with 4–6 repetitions)	6 weeks (1 session per week)	Non-specific sport movement	Improvement in countermovement jump performance
Fiorilli et al. (2020b) [36]	12 male soccer players aged 13.3 ± 0.7	4 m multidirectional sprint (4 sets with 10 repetitions)	Single session (acute)	Specific sport movement	Improvement in squat jump, countermovement jump, and drop jump performance
Raya-González et al. (2021b) [37]	20 male soccer players with age < 16	Lateral squat (2–4 sets with 8–10 repetitions)	10 weeks (1 session per week)	Non-specific sport movement	Improvement in countermovement jump performance
De Hoyo et al. (2015b) [38]	20 male soccer players aged 17.0 ± 1.0	Half squat (4 sets with 6 repetitions)	Single session (acute)	Non-specific sport movement	Improvement in countermovement jump performance
Stojanović et al. (2021) [40]	36 male basketball players aged 17.58 ± 0.5	Romanian deadlift and half squats (2–4 sets with 8 repetitions)	8 weeks (1–2 sessions per week, for a total of 12 training session)	Specific and non-specific sport movement	Improvement in countermovement jump
Cabanillas et al. (2020) [41]	8 male basketball players aged 21.3 ± 3.45	Half squat (4–6 sets with 10 repetitions)	6 weeks (1 session per week)	Specific and non-specific sport movement	Improvement in countermovement jump
Nunez et al. (2018) [42]	27 athletes practicing team sports aged 22.7 ± 2.8	Unilateral lunge and bilateral squat (4 sets with 7 repetitions)	6 weeks (2 sessions per weeks)	Non-specific sport movement	Improvement in countermovement squat jump
Westblad et al. (2021) [43]	25 male and female athletes practicing team sports aged 11.8 ± 0.9	Squat (4 sets with 6 repetitions)	6 weeks (2 sessions per week)	Non-specific sport movement	Improvement of squat jump performance, but no difference compared to control group
di Cagno et al. (2020) [45]	54 male fencers aged 17.4 ± 2.3	Forward and backward lunge, two steps forward and backward, simulating the fencing assault (3–4 sets with 7–9 repetitions)	6 weeks (2 per week)	Non-specific sport movement	No improvement in jump performance
Canós et al. (2021) [46]	24 male tennis players aged 15.7 ± 1.1	Low row 90°, forehand closed stance, backhand closed stance, one-handed chest crossover, one-handed low row, chest press, one-handed shoulder press (3 sets with 6–8 repetitions)	8 weeks (2 sessions per week)	Specific and non-specific sport movement	Improvement in countermovement jump performance; greater improvement in the first 4 weeks
Sabido et al. (2017) [48]	18 male handball players aged 23.9 ± 3.8	Bilateral half squat (4 sets with 8 repetitions) and unilateral lunge (2 sets with 8 repetitions)	7 weeks (1 session per week)	Non-specific sport movement	Improvement in triple hop distance performance

**Table 6:** Effect of flywheel training on change of direction (CoD) performance.

Authors (Reference No.)	Sport and Sample	Training Performed	Duration and Frequency of Training Sessions	Specific or Not Specific Sport Movement	Main Results Obtained
Fiorilli et al. (2020a) [21]	34 male soccer players aged 13.28 ± 1.0	Multidirectional 4 m sprint, shot movement (4 sets with 7 repetitions)	6 weeks (2 sessions per week)	Specific sport movement	Improvement in CoD performance
Coratella et al. (2019) [32]	40 male soccer players aged 23 ± 4	Squat (4–6 sets with 8 repetitions)	8 weeks (1 session per week)	Non-specific sport movement	Improvement in CoD performance
Fiorilli et al. (2020b) [36]	12 male soccer players aged 13.3 ± 0.7	4 m multidirectional sprint (4 sets with 10 repetitions)	Single session (acute)	Specific sport movement	Improvement in CoD performance that persisted up to 10 min after administration of the warm-up
Raya-González et al. (2021b) [37]	20 male soccer players with age < 16	Lateral squat (2–4 sets with 8–10 repetitions)	10 weeks (1 session per week)	Non-specific sport movement	Improvement in CoD performance
De Hoyo et al. (2015b) [38]	20 male soccer players aged 17.0 ± 1.0	Half squat (4 sets with 6 repetitions)	Single session (acute)	Non-specific sport movement	Improvement in contact time and force during CoD performance
Stojanović et al. (2021) [40]	36 male basketball players aged 17.58 ± 0.5	Romanian deadlift and half squat (2–4 sets with 8 repetitions)	8 weeks (1–2 sessions per week, for a total of 12 training session)	Specific and non-specific sport movement	Improvement in CoD task compared with traditional basketball training
Nunez et al. (2018) [42]	27 athletes practicing team sports aged 22.7 ± 2.8	Unilateral lunge and bilateral squat (4 sets with 7 repetitions)	6 weeks (2 sessions per weeks)	Non-specific sport movement	Improvement in CoD, with angles of 90°

## Data Availability

The data presented in this study are available upon request from the corresponding author.
